# The Influence of Gender on the Choice of Radiology as a Specialty Among Medical Students in Saudi Arabia: Cross-Sectional Study

**DOI:** 10.2196/14666

**Published:** 2020-04-28

**Authors:** Ahmed H Abduljabbar, Sara F Alnajjar, Hussein Alshamrani, Lujain F Bashamakh, Hisham Z Alshehri, Waleed M Alqulayti, Mohammad A Wazzan

**Affiliations:** 1 Department of Radiology King Abdulaziz University Hospital Jeddah Saudi Arabia; 2 College of Medicine King Abdulaziz University Jeddah Saudi Arabia

**Keywords:** gender, radiology, specialty choice

## Abstract

**Background:**

Medical undergraduates are the future doctors of the country. Therefore, determining how medical students choose their areas of specialty is essential to obtain a balanced distribution of physicians among all specialties. Although gender is a significant factor that affects specialty choice, the factors underlying gender differences in radiology are not fully elucidated.

**Objective:**

This study examined the factors that attracted medical students to and discouraged them from selecting diagnostic radiology and analyzed whether these factors differed between female and male medical students.

**Methods:**

This cross-sectional study conducted at King Abdulaziz University Hospital in Jeddah, Saudi Arabia, used an electronic questionnaire sent to medical students from all medical years during February 2018. Subgroup analyses for gender and radiology interest were performed using the chi-square test and Cramér’s V test.

**Results:**

In total, 539 students (276 women; 263 men) responded. The most common factor preventing students from choosing radiology as a career was the lack of direct patient contact, which deterred approximately 47% who decided against considering this specialty. Negative perceptions by other physicians (*P*<.001), lack of acknowledgment by patients (*P*=.004), and lack of structured radiology rotations (*P*=.007) dissuaded significantly more male students than female students. Among those interested in radiology, more female students were attracted by job flexibility (*P*=.01), while more male students were attracted by focused patient interactions with minimal paperwork (*P*<.001).

**Conclusions:**

No significant difference was found between the genders in terms of considering radiology as a specialty. Misconception plays a central role in students’ judgment regarding radiology. Hence, early exposure to radiology, assuming a new teaching method, and using a curriculum that supports the active participation of students in a radiology rotation are needed to overcome this misconception.

## Introduction

According to the latest statistics from the Saudi Commission for Health Specialties, there are 37 medical colleges throughout Saudi Arabia, and 4042 students are expected to graduate in 2020. Among these students, 1781 are female, and 2261 are male [1]. Medical undergraduates are the future doctors of the country; therefore, determining how medical students choose their areas of specialty is essential to obtain a balanced distribution of physicians among all specialties [2].

Factors including personal interactions, lifestyle choices, society’s perception, high financial status, job opportunities, and interest in research have been found to influence the selection of a medical specialty [3-5]. In Saudi Arabia, previous studies stated that medical students were influenced to choose a medical career mostly by a flexible lifestyle, high income, and prestige [6,7].

Gender is another significant factor that affects specialty choice. Female students are more likely to favor job flexibility (namely, the option to work part-time) and the lifestyle [8,9], while male students are more concerned about technical challenges, society’s perception, and learning potential [10].

In fact, the total number of female medical students has increased to that of male medical students. However, there are fewer female students in some medical specialties, such as diagnostic radiology [11-13]. The factors discouraging female students from choosing diagnostic radiology are similar to those in other medical careers [14].

A previous study conducted to find the reasons for choosing radiology as a career among radiologists found 4 important factors: interest in diagnostic radiology, quality of life, variety of practice, and fixed work hours [11]. A greater proportion of female radiologists (60%) than male radiologists (43%) took into consideration the work hours and believed it was the main reason they decided to join the radiology field. These findings are understandable since women need more flexible working shifts than do men during their child-rearing years [15].

One study showed that indirect patient care and nonclinical work were the major factors that dissuaded American clerkship students from choosing diagnostic radiology as a career [16]. However, evidence suggests that good exposure during medical education may help students choose diagnostic radiology as a career in the future [11].

The factors underlying gender differences in radiology are not fully elucidated. The purpose of this research was to examine the factors that attract medical students to and discourage them from selecting diagnostic radiology as a specialty and to analyze whether these factors differ between female and male medical students in Saudi Arabia.

## Methods

This cross-sectional study was conducted at King Abdulaziz University Hospital (KAUH) in Jeddah, Saudi Arabia. Ethical approval for the study was provided by the Institutional Review Board of KAUH.

We included participants from all medical years. The study was conducted by sending an electronic questionnaire to the medical students of KAUH during February 2018. The students were given 1 month to return their reply after receiving the questionnaire. In the meantime, we sent 2 reminder emails about the questionnaire. The survey was sent to 1127 students, and only 539 (47.8%) answered the questionnaire.

The questionnaire was obtained from a previously published study [17]. It consisted of 4 sections. The first section collected information about any previous radiology exposure or any mentorship the respondent had received in the radiology field. The second section collected information about any factors that affected their specialty choice. The third section collected information about the factors that discouraged them from choosing radiology as a career. This section was to be answered only by students who were not interested in radiology. The fourth section collected information about the factors that encouraged them to choose radiology as a career. This section was to be answered only by students who were considering radiology as a career. In the “previous radiology exposure” section, a preclinical observership, wherein a student attended radiology out of personal interest, was not a mandatory requirement before clerkship. An elective was defined as a rotation in which a student could select any specialty and he/she selected radiology. The answers were in a “select all that apply” format.

The data were entered into Microsoft Excel (Microsoft Corporation, Redmond, WA) and then transferred to IBM SPSS Statistics for Windows, Version 23.0 (IBM Corp, Armonk, NY) for further analysis. Categorical variables, including primary variables, were described using a frequency table and subsequently processed to calculate the statistical significance using the chi-square test and Cramér’s V test. For all statistical tests, *P* values <0.05 were considered significant.

## Results

A total of 539 students participated, resulting in a response rate of 47.8% (539/1127). Of these students, 276 were female (276/539, 51.2%), and 263 were male (263/539, 48.8%). In the total sample, 266 (266/539, 49.3%) students were in their pre-clerkship years, and 273 (273/539, 50.6%) students were in their clerkship years. No significant difference was observed between genders in the level of training. Students who were potentially considering radiology as a career numbered 83 (83/539, 15.4%), while 456 (456/539, 84.6%) were not interested in considering radiology as a career. Among the students interested in radiology, 40 were female (40/83, 48.2%), and 43 were male (43/83, 51.8%). No significant difference was found between genders regarding the consideration of radiology as a specialty.

### Radiology Exposure

Among the students considering radiology as a career, more men had a radiology mentor than did women (*P*<.001). In addition, more male students conducted radiology-related research than did female students (*P*<.001), but no gender-specific differences were observed in mentorship and research experience among those not interested in radiology. Among the students not interested in radiology, more men had received didactic lectures than did women (*P*=.01; Table 1).

**Table 1:** Method of exposure to radiology, by gender and interest in the specialty.

Previous radiology exposure	Considering radiology, n (%)			Not considering radiology, n (%)		
	Female students (n=40)	Male students (n=43)	*P* value	Female students (n=236)	Male students (n=220)	*P* value
A. None	12 (30.0)	10 (23.3)	.65	99 (41.9)	104 (47.3)	.29
B. Preclinical observerships	7 (17.5)	5 (11.6)	.65	36 (15.2)	39 (17.7)	.58
C. Preclinical didactic lectures	11 (27.5)	10 (23.3)	.84	81 (34.3)	53 (24.0)	.01
D. Radiology research experience	6 (15.0)	22 (51.2)	<.001	3 (1.3)	5 (2.3)	.69
E. Core rotations in clerkship	7 (17.5)	12 (27.9)	.38	57 (24.2)	45 (20.5)	.19
F. Elective rotations in clerkship	12 (30.0)	8 (18.6)	.33	9 (3.8)	7 (3.2)	.91
G. Radiology mentor	0 (0)	10 (23.2)	.001	3 (1.3)	7 (3.2)	.20
H. Radiologist family member	4 (10.0)	5 (11.6)	>.99	14 (5.9)	9 (4.1)	.49
I. Attended a radiology conference	3 (7.5)	8 (18.6)	.24	7 (3.0)	11 (5.0)	.38

### Specialty Choice

More male students not considering radiology as a specialty rated direct patient contact (*P*=.003) and impact on patient care (*P*=.01) as important factors than did those interested in radiology. In contrast, more male students who were interested in radiology as a career were attracted by fewer working hours (*P*=.03), job flexibility (*P*=.008), and fewer years of training (*P*=.02) than were those not interested in radiology.

A greater proportion of female students interested in radiology as a career (23/40, 57.5%) were attracted by high income than female students not interested in the specialty (92/236, 38.9%; *P*=.02). In addition, more female students not interested in radiology rated the impact on patient care (116/236, 49.1%; *P*=.008), job opportunities (98/236, 41.5%; *P*=.04), and use of emerging technology (64/236, 27.1%; *P*=.005) as important than did female students interested in radiology. Factors that influenced the choice of a medical specialty are shown in Table 2.

**Table 2:** Factors affecting specialty choice, by gender and interest in radiology.

Factor	Female students, n (%)			Male students, n (%)		
	Not considering radiology (n=236)	Considering radiology (n=40)	*P* value	Not considering radiology (n=220)	Considering radiology (n=43)	*P* value
A. High income	92 (38.9)	23 (57.5)	.02	127 (57.7)	28 (65.1)	.46
B. Fewer working hours	61 (25.8)	16 (40.0)	.09	71 (32.2)	21 (48.8)	.03
C. Job flexibility	90 (38.1)	19 (47.5)	.34	80 (36.0)	25 (58.0)	.008
D. Intellectual stimulation	58 (24.5)	5 (12.5)	.13	59 (26.8)	9 (20.9)	.53
E. Use of emerging technology	64 (27.1)	2 (5.0)	.005	43 (19.5)	12 (27.9)	.30
F. Direct patient contact	102 (43.2)	13 (32.5)	.27	82 (37.0)	6 (13.9)	.003
G. Impact on patient care	116 (49.1)	10 (25.0)	.008	82 (37.3)	7 (16.3)	.01
H. Perception by others	14 (5.9)	2 (5.0)	>.99	30 (13.6)	5 (11.6)	.91
I. Job satisfaction	140 (59.3)	20 (50.0)	.35	132 (60.5)	22 (51.2)	.33
J. Available job opportunities	98 (41.5)	10 (25.0)	.04	84 (38.2)	16 (37.2)	>.99
K. Fewer years of residency	34 (14.4)	2 (5.0)	.16	23 (10.4)	10 (23.3)	.02
L. Research opportunities	41 (17.3)	3 (7.5)	.16	40 (18.2)	6 (14.0)	.65
M. Positive training experience	70 (29.6)	11 (27.5)	.92	44 (20.0)	6 (14.0)	.47
N. Positive mentorship experience	50 (21.1)	5 (12.5)	.29	44 (20.0)	4 (9.3)	.14
O. Favorable to having children	45 (19.0)	7 (17.5)	.98	33 (15.0)	9 (20.9)	.40

### Factors Attracting Students to Radiology

The top factors attracting medical students to radiology as a career included focused patient interactions with minimal paperwork, interest in anatomy, and job flexibility (Table 3). More female students (Cramér’s V=0.12, *P*=.01) were attracted by the option to work part-time (namely, job flexibility), while more male students were attracted by focused patient interactions with minimal paperwork (Cramér’s V=0.4, *P*<.001).

**Table 3:** Factors enticing medical students to radiology, among those considering radiology as a career.

Factor	Male students (n=43), n (%)	Female students (n=40), n (%)	*P* value
A. Physics knowledge	12 (27.9)	13 (32.5)	.83
B. Interest in anatomy	13 (30.2)	19 (47.5)	.17
C. Wide range of medical knowledge	13 (30.2)	14 (35.0)	.82
D. Role as a consultant to other doctors	7 (16.2)	10 (25.0)	.48
E. Having a task-based workday	13 (30.2)	14 (35.0)	.82
F. Focused patient interactions with minimal paperwork	28 (65.1)	1 (2.5)	**<**.001
G. Impact on patient care	9 (20.9)	10 (25.0)	.86
H. High income	15 (34.9)	13 (32.5)	>.99
I. Positive perception of radiology by others	4 (9.3)	2 (5.0)	.67
J. Positive prior exposure to radiology as a specialty	5 (11.6)	9 (22.5)	.30
K. Positive radiology mentorship experience	4 (9.3)	3 (7.5)	>.99
L. Job flexibility (ie, opportunity to work part-time)	12 (27.9)	23 (57.5)	.01
M. Passionate to be an interventional radiologist	9 (20.9)	10 (25.0)	.86
N. Intellectual stimulation	8 (18.6)	7 (17.5)	>.99
O. Interest in radiology research	5 (11.6)	5 (12.5)	>.99
P. Perceived availability of job opportunities	12 (27.9)	16 (40.0)	.35

### Factors Dissuading Students From Radiology

The most common factor that dissuaded students from choosing radiology as a career was the lack of direct patient contact, which deterred 43.2% (95/220) of male students and 50.8% (120/236) of female students who had decided not to consider radiology as a specialty (Table 4). For each gender, among female students, the second most common factor was potential exposure to radiation (88/236, 37.7%), while among males, the second most common factor was a negative perception by other physicians (71/220, 32.1%).

A negative perception by other physicians (Cramér’s V=0.8, *P*<.001), lack of acknowledgment by patients (Cramér’s V=0.13, *P*=.004), and lack of structured radiology rotations (Cramér’s V=0.12, *P*=.007) dissuaded significantly more male students than female students, as shown in Table 4.

**Table 4:** Factors dissuading medical students from radiology among those not considering radiology, by gender.

Factor	Male students (n=220), n (%)	Female students (n=236), n (%)	*P* value
A. Physics knowledge	43 (19.5)	55 (23.3)	.39
B. Role as a consultant to other doctors	16 (7.2)	24 (10.2)	.35
C. Lack of direct patient contact	95 (43.2)	120 (50.8)	.12
D. Negative perception by other doctors	71 (32.1)	8 (3.3)	**<**.001
E. Lack of acknowledgment by patients	52 (23.6)	30 (12.7)	.004
F. Possible exposure to radiation	65 (29.5)	88 (37.7)	.09
G. Competitiveness in attaining a residency position	14 (6.3)	19 (8.0)	.61
H. Lack of prior exposure to radiology as a specialty	32 (14.5)	28 (11.9)	.48
I. Lack of structured radiology rotations and courses	32 (14.5)	15 (6.4)	.007
J. Lack of radiology mentorship	15 (6.8)	16 (6.8)	>.99
K. Perceived lack of working part-time	8 (3.6)	7 (3.0)	.89
L. Perceived lack of job satisfaction	34 (15.3)	36 (15.3)	>.99
M. Perceived lack of job opportunities	18 (8.1)	13 (5.5)	.34
N. Already pursuing another specialty	65 (29.5)	78 (33.1)	.48
O. Bad personal experience in radiology	18 (8.1)	9 (3.8)	.07
P. Working in a dark environment	34 (15.5)	52 (22.0)	.09
Q. Perception as a male-dominated career	4 (1.8)	7 (3.0)	.62
R. Lack of research support or opportunities	11 (5.0)	16 (6.8)	.45
S. Lack of procedures performed by non-interventional radiologists	12 (5.5)	14 (5.9)	.99

## Discussion

The aim of this study was to scrutinize the factors that attracted medical students to and deterred them from selecting diagnostic radiology as a specialty and to analyze whether these factors vary between male and female students. In this survey, compared with the percentage of male students, the percentage of female students considering radiology as a specialty was inconsistent with that in the literature. In this study, the numbers of female and male participants interested in radiology were almost the same, while the literature suggests that female participants consider radiology less frequently than do male participants [17-20]. The female lifestyle in Saudi Arabia and the great attention they pay to child rearing, with considerable concern for their family in general, could be the cause for this disparity. Our finding agrees with that in a previous survey carried out in Saudi Arabia, which concluded that marital status, which is an important part of social life, was a considerable predictor for job selection among female participants [21].

Of the 16 attractive factors that we studied, the most important factor that attracted female participants to radiology was job flexibility. This was emphasized by another study conducted in Canada [17]. In the SwissMedCareer Study, the percentage of female physicians who had children and worked full-time was only 18.3% [22]. Furthermore, when radiology residents were asked about the factors they think would attract medical students to consider radiology, they reported the ability to work part-time and maintain work-family harmony as the 2 most important factors [23]. An American study demonstrated that 60% of women and only 7% of men were working part-time (*P*<.01) [24]. This result is unsurprising as women seek more balance between work and life, including child-care, which is considered the main reason women consider part-time work [23,25]. In summary, a controllable lifestyle has a remarkable influence on medical students’ specialty preferences and, therefore, has the potential to attract female students towards radiology [11,26]. However, women in diagnostic radiology are unequally represented within radiology residency training programs compared with other residency training programs [27]. For male students, the most attractive part of radiology is the focused patient interaction with minimal paperwork. This could be because male students prefer performing a focused task rather than being responsible for multiple tasks.

The most crucial factor that dissuaded both male and female students from considering radiology as a specialty was the lack of direct patient contact. This is concordant with the findings of other studies that confirmed this as the most significant factor [18,19,28,29]. However, female students concentrate on some subspecialties and overlook others, including interventional radiology, that enable the most patient contact [11,17,30]. There are two probable explanations for this. First, female students have insufficient information about interventional radiology; a Saudi study emphasized that more than half of respondents believed that they had poor or no information about this subspecialty [31]. Second, female students avoid this subspecialty because it is less flexible, lacks the opportunity to work part-time, and is associated with long hours and physically demanding work [17], which they considered an extremely important characteristic when selecting radiology as a career. To solve the issue of women not considering a subspecialty such as interventional radiology, a program should be initiated that encourages the early exposure of students to subspecialties that involve direct contact with patients, and the available opportunities for patient contact in these subspecialties should be highlighted [18,28]. For male students specifically, the lack of acknowledgement by patients was ranked fourth among the choices. This outcome can be interpreted based on the lack of direct contact with the patient, which obviates patient appreciation. Nevertheless, highlighting the significant role of radiology in treating patients and improving their health could help overcome this notion.

The second most common factor that dissuaded female students from selecting radiology as a specialty was potential exposure to radiation. A fear of cancer, specifically breast cancer, could account for this; breast cancer is the most common cancer in women, and radiation exposure is a risk factor [32,33]. For male students, the second factor was a negative perception by other physicians. Notably, this specific reason was also the second most important deterrent among male participants in another study [28]. Many reasons could inculcate this thought, including that the decision to pursue radiology can be influenced by intensive radiology exposure [27]. We think that limited exposure to radiology may have a role in the development of this negative perception among students. Compared with other specialties that are studied in detail and over a long period of time, students of radiology are involved in a very short rotation with only a few scattered lectures throughout the 6 academic years and few available courses. This concept supports another of our findings, namely that a lack of structured rotations and selective courses dissuaded more male students from this specialty. Furthermore, as a result of limited exposure, radiologists will not have enough time to wholly and effectively display their expertise. The manner in which doctors from different specialties introduce radiology to students during classes and rounds could perhaps have an effect. However, it is unclear why this factor was specifically mentioned by male students.

Having a passion for another specialty was one of the top 3 dissuading factors among students of both genders. At our institution, the radiology rotation is presented to the students in the fourth year, a time at which most are already passionate about another specialty [20] and a short rotation will probably not change their choice. Therefore, radiology should be introduced to medical students in their first years, and it must be taught in a way that encourages active participation rather than just observing.

Overall, specialty choice was more affected by direct patient contact and impact on patient care for male respondents preferring non-radiology specialties. Students who are concerned about patient contact and patient care seem less interested in radiology and vice versa [34]. It must be emphasized that radiology has a prominent role in patient care since most patients undergo radiological imaging as a part of their diagnostic journey [28]. Further, the process of image interpretation is not a separate process from patient care, and in fact, it is influenced by the patient’s history and presentation [18]. Therefore, patient care is an integrated system where success depends on the contribution of many physicians from different specialties with a final goal of saving patients’ lives. Among students interested in radiology as a career, when compared with students not interested, the male students’ choices were more affected by fewer working hours, job flexibility, and fewer years of training, while the female students’ choices were influenced by a high income. These findings are supported by those of another study, which showed that specialty choice has recently been influenced by many factors, including a controllable lifestyle and high income [26].

Male students interested in radiology also conducted more research activities in radiology than did female students. This finding is consistent with that of a Canadian study [17]. Additionally, more male students interested in radiology had a radiology mentor than did female students. In fact, the presence of a mentor is considered an essential factor that affects specialty choice [18,35].

Although the aim of the study was achieved, there were some limitations. Since the main topic of the study was the specialty of radiology, bias may be present because students who are considering radiology as a career may be more interested than other students in answering the questionnaire. Furthermore, almost half of the students were in the pre-clerkship years. As a result, their exposure and knowledge about radiology and other specialties could be restricted. In fact, we did not assess the students’ career choice after graduation, and we only assessed their interest in radiology in general. Moreover, this survey did not illustrate whether exposure to radiology would markedly increase the students’ interest in radiology. Thus, a study analyzing the students’ opinion before and after exposure to the specialty of radiology is warranted. Finally, this was a cross-sectional study that cannot determine causality between the studied factors and the choice of the radiology specialty.

### Conclusion

Many factors could influence the decision of medical students to consider diagnostic radiology as a career in the future. The present work has shown that the most discouraging factor is the lack of direct patient contact, whereas the most encouraging factor is job flexibility for female students and focused patient interactions with minimal paperwork for male students. Furthermore, no significant difference was found between the genders related to considering radiology as a specialty. We observed that misconception plays a central role in students’ judgments regarding radiology specialties. Hence, early exposure to radiology, assuming a new teaching method, and using a curriculum that supports the active participation of students in a radiology rotation are needed to overcome this misconception.

## Abbreviations

KAUH: King Abdulaziz University Hospital
